# An Efficient *Agrobacterium*-Mediated Transformation of Strawberry cv. Camarosa by a Dual Plasmid System

**DOI:** 10.3390/molecules20033647

**Published:** 2015-02-23

**Authors:** Fatemeh Haddadi, Maheran Abd Aziz, Siti Nor Akmar Abdullah, Soon Guan Tan, Hossein Kamaladini

**Affiliations:** 1 Department of Agriculture Technology, Faculty of Agriculture, Universiti Putra Malaysia, Serdang, Selangor 43400, Malaysia; E-Mails: snaa@upm.edu.my (S.N.A.A.); hu_kamaladiny@yahoo.com.au (H.K.); 2 Laboratory of Plantation Crops, Institute of Tropical Agriculture, Universiti Putra Malaysia, Serdang, Selangor 43400, Malaysia; 3 Department of Cell and Molecular Biology, Faculty of Biotechnology and Biomolecular Sciences, Universiti Putra Malaysia, Serdang, Selangor 43400, Malaysia; E-Mail: sgtan@upm.edu.my; 4 Department of Biology, Faculty of Sciences, University of Zabol, Zabol 9861335856, Iran

**Keywords:** stable transformation, *Agrobacterium*, strawberry camarosa, pGreen0049 binary vector, thidiazuron, timentin, cefotaxime

## Abstract

An *Agrobacterium-*mediated transformation method was applied to introduce the luciferase reporter gene under the control of the CaMV35S promoter in the pGreen0049 binary vector into strawberry cv. Camarosa. The *in vitro* regeneration system of strawberry leaves to be used in the transformation was optimized using different TDZ concentrations in MS medium. TDZ at 16 µM showed the highest percentage (100%) of shoot formation and the highest mean number of shoots (24) produced per explant. Studies on the effects of different antibiotics, namely timentin, cefotaxime, carbenicillin and ampicillin, on shoot regeneration of strawberry leaf explants showed the best shoot regeneration in the presence of 300 mg/L timentin and 150 mg/L cefotaxime. Assessment of the different factors affecting *Agrobacterium* mediated-transformation of strawberry with the luciferase gene showed the highest efficiency of putative transformant production (86%) in the treatment with no preculture, bacterial OD_600_ of 0.6 and the addition of 150 mg/L cefotaxime in the pre-selection and selection media. The presence of the luciferase gene in the plant genome was verified by the luciferase reporter gene assay, nested PCR amplification and dot blot of genomic DNA isolated from the young leaves of each putatively transformed plantlet.

## 1. Introduction

There is worldwide growing demand for strawberries due to their richness in vitamins, polyphenolics and phytochemicals, pleasant aroma and flavor, and fresh appearance. Strawberries constitute an important component of products obtained through greenhouse production, and many studies have been conducted on the species. Cultivated strawberry (*Fragaria x ananassa* Duch.) a member of the *Rosaceae* family, was first introduced in the 1750s. Presently, there are more than twenty *Fragaria* species representing many levels of ploidy within the genus that vary remarkably in size, color, flavor, shape, degree of fertility, season of ripening, susceptability to disease and constitution of plant [1]. Intraspecific crosses of *F.* × *ananassa* (8×) to improve its agronomic traits resulted in the introduction of new cultivars. The octoploid strawberry cv. Camarosa is one of the most important commercial cultivars cultivated in Malaysia. The complicated genetic background due to high heterozygosity and polyploidy of this cultivar impedes improvement of this crop through traditional breeding methods. Manipulation at the gene level paves the way for further improvement of agronomic traits of strawberry cv. Camarosa.

An efficient plant regeneration system is a precondition for the development of an *Agrobacterium*-mediated transformation protocol for cultivated strawberry species. Strawberry leaf is among the most popularly tested explants for regeneration. In spite of the impressive regeneration system from leaf explant for some commercial genotypes, the regeneration of putatively transformed cells is strictly influenced by genotype [2]. Therefore transformation of strawberry is still inefficient and often not repeatable [3,4,5,6] requiring 40–112 days for the emergence of the first visible shoots [7].

Moreover, different antibiotics used for suppressing *Agrobacterium* growth in plant transformation have different effects on the regeneration abilities of the various plant tissues. The appropriate choice of an antibiotic and its concentration for transformation can ameliorate the problem of poor transformation and chimerism [8]. In addition to carbenicillin and cefotaxime commonly used [9,10], timentin is another effective antibiotic for the suppression of *Agrobacterium* in many plant transformation systems [11]. In this study the effects of different concentrations of thidiazuron (TDZ) and four different antibiotics, namely timentin, cefotaxime, carbenicillin and ampicillin, were evaluated on *in vitro* shoot regeneration from the leaves of strawberry cv. Camarosa.

Besides *in vitro* regeneration as an important factor affecting the efficiency of stable transformation [12], explant wounding, bacterial strains, co-cultivation duration, acetosyringone, vector, selective marker and competency of target plant tissues for infection are other factors that need to be considered [13,14,15,16,17,18,19].

Hence this research was conducted to optimize factors that affect *Agrobacterium*-mediated transformation of strawberry cv. Camarosa using recombinant pGreen0049 vector harbouring a luciferase reporter gene under the control of the CaMV35S promoter by a dual plasmid system pGreen/pSoup [20], followed by verification of the presence of the luciferase gene in the plant genome.

## 2. Results and Discussion

### 2.1. Effects of Different Concentrations of TDZ on Shoot Regeneration of Strawberry Leaves

Shoot regeneration began at the cut sides and adaxial surface of the leaf explants in the presence of 12, 16 and 24 µM TDZ after three weeks of culture and subsequently the number and length of shoots increased until the fifth week of culture. No regeneration was observed on the medium without TDZ. Regeneration occurred as well on the abaxial side of the leaves in 12, 16, 24 and 32 µM of TDZ. Meanwhile for the concentrations of 4 and 8 µM TDZ the leaf explants only formed callus on the abaxial side.

**Figure 1:**
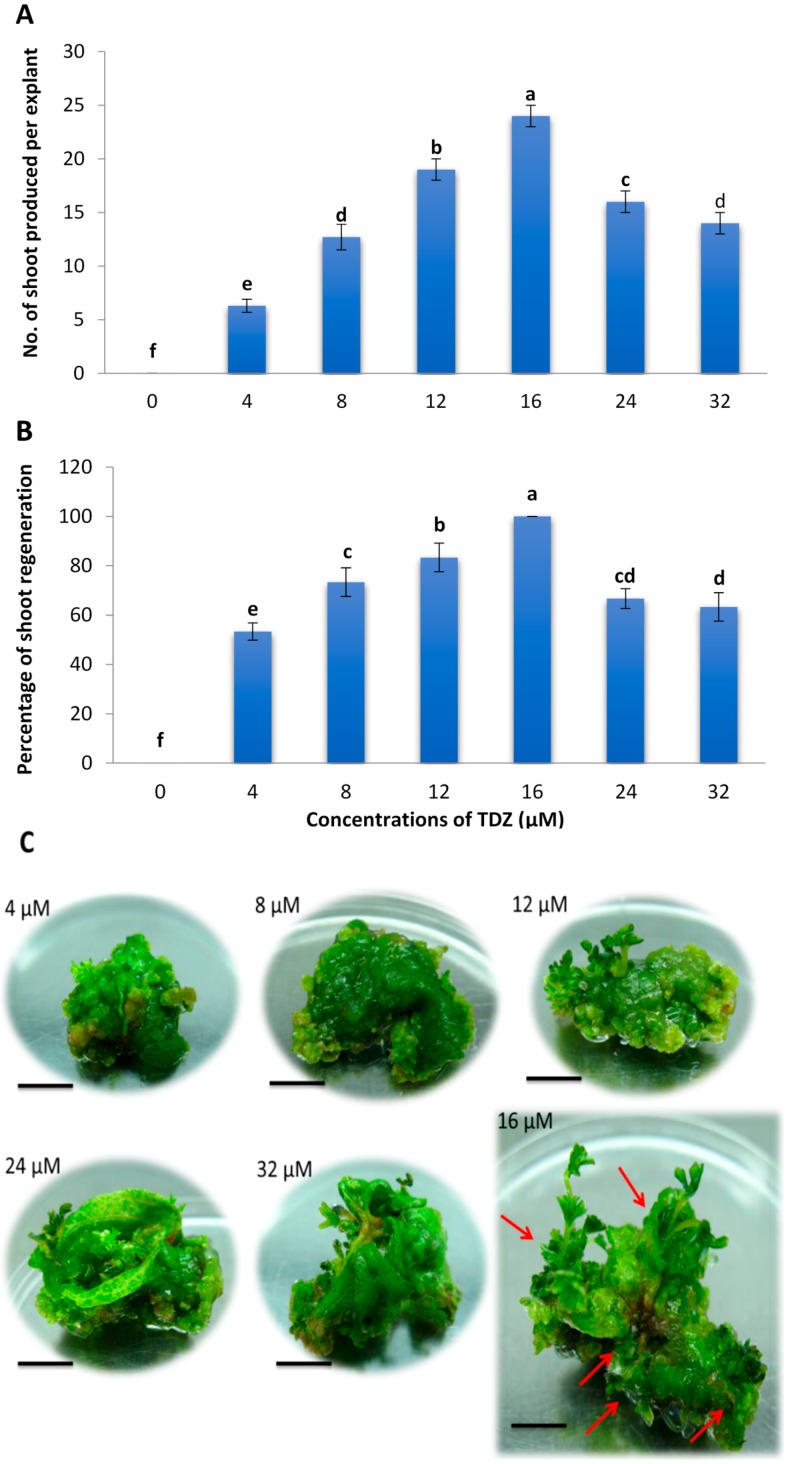
Effects of different concentrations of TDZ on: (**A**) mean number of shoots produced per leaf explant; (**B**) percentage of shoot formation; and (**C**) Shoot regeneration from leaf explant of strawberry cv. Camarosa after five weeks of culture. Arrows show the regenerated shoots (Bars = 1 cm). Means followed by the same letter (s) are not significantly different based on DMRT.

The number and percentage of shoots produced per explant were significantly affected by the different concentrations of TDZ tested. A moderate concentration of 16 µM TDZ showed the highest number of shoots produced per explant (24) and the highest percentage of shoot formation (100%) (Figure 1A,B). Increasing the concentration of TDZ from 0 to 16 µM increased the shoot regeneration number and percentage but they were reduced by increasing the concentration from 16 to 32 µM. Figure 1C shows shoot regeneration from strawberry leaf explants in the presence of different TDZ concentrations.

### 2.2. Effects of Different Antibiotics on Shoot Regeneration from Strawberry Leaves

Different antibiotics have different phytotoxic effects on shoot regeneration from the leaf explants. Shoot regeneration started after three weeks of culture in the control and treatments with antibiotic. Among all the tested antibiotics, carbenicillin showed the high callus formation while timentin was similar to the control with the least amount of callus formation on the leaves.

**Figure 2:**
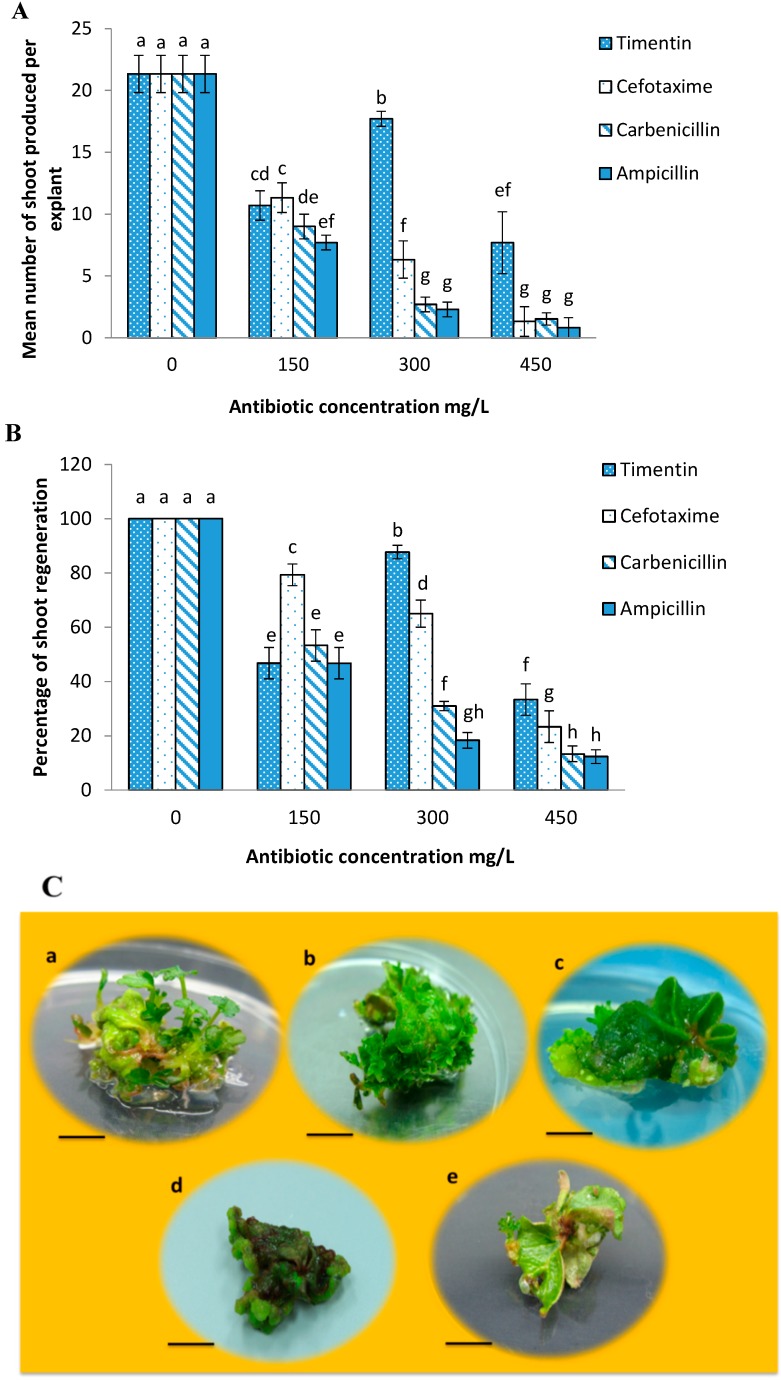
Effects of different concentrations of timentin, cefotaxime, carbenicillin and ampicillin on: (**A**) mean number of shoots produced per explant; (**B**) percentage of shoot regeneration from leaf explant; and (**C**) Shoot regeneration from leaf explant of strawberry cv. Camarosa after five weeks of culture. a: Control (without antibiotic); b: 300 mg/L timentin; c: 150 mg/L cefotaxime; d: 150 mg/L carbenicillin and e: 150 mg/L ampicillin (Bars = 1 cm). Means followed by the same letter (s) are not significantly different based on DMRT.

The results of this study showed significant differences among timentin, cefotaxime, carbenicillin and ampicillin on shoot regeneration from strawberry leaves. The highest mean number of shoot regeneration (21.33) and the highest percentage of shoot regeneration (100%) were attained on antibiotic free MS medium as a control (Figure 2). The highest mean number of shoots produced per explant for cefotaxime, carbenicillin and ampicillin was 13, 10.5 and 8 in the presence of 150 mg/L of each antibiotic respectively which were significantly lower when compared to the control. As for timentin, 300 mg/L timentin produced the highest mean number of shoots per explant (18.3) which was lower than the control but significantly higher than the other tested antibiotics (Figure 2A). Indeed timentin had less phytotoxic effects on shoot regeneration even at higher concentrations when compared to the other three antibiotics.

In addition, the highest percentage of shoot production 80%, 55%, 50% obtained on medium with 150 mg/L of each antibiotic, cefotaxime, carbenicillin and ampicillin respectively were significantly lower compared to the control. For timentin, the highest percentage of shoot regeneration was 88% at the concentration of 300 mg/L (Figure 2B). On MS medium containing cefotaxime, carbenicillin and ampicillin respectively, increasing their concentrations from 150 to 450 mg/L caused reduction in the mean number and percentage of shoot regeneration. Meanwhile for timentin, there was an increase in the mean number and percentage of shoot regeneration up to 300 mg/L (Figure 2A,B). Subsequently, timentin at 300 mg/L and cefotaxime at 150 mg/L were selected for inclusion in the preselection and selection media after *Agrobacterium*-mediated transformation. Figure 2C shows the shoot regeneration occurring in the presence of different antibiotics.

### 2.3. Minimum Inhibitory Concentration of Kanamycin on Leaf Explant

**Figure 3:**
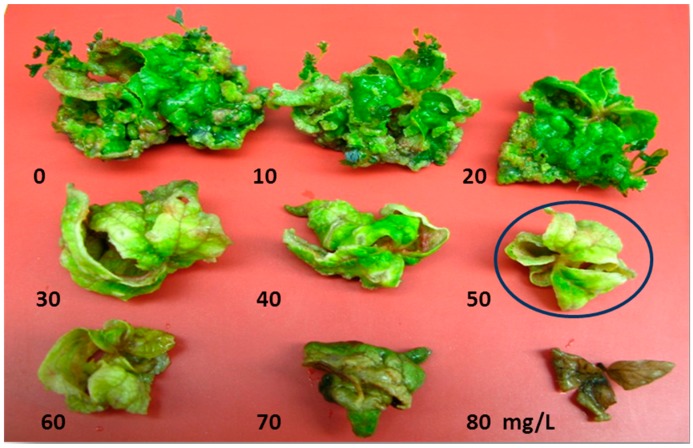
Minimum lethal dosage of kanamycin on shoot regeneration from strawberry cv. Camarosa leaf explant after five weeks of culture. Kanamycin at the concentration of 50 mg/L was selected as the MIC for strawberry leaf (Bar = 1 cm).

Shoot regeneration started after 20 days for control treatment without kanamycin and after 24 days in treatments with 10 and 20 mg/L of kanamycin. Shoots were formed on leaf explants cultured on 30 and 40 mg/L kanamycin only after one month of culture. The highest percentage of shoot regeneration (100%) was obtained on MS medium with no addition of kanamycin and the minimum percentage of 0% was achieved on MS medium at 50 mg/L kanamycin or higher concentration (Table 1). Meanwhile, leaves cultured on 50 mg/L kanamycin do not form shoot even after five weeks of culture, therefore that concentration was selected as the MIC for effective screening of putative transformants after subjecting leaf explant to *Agrobacterium-*mediated transformation. The leaves cultured on 60 and 70 mg/L kanamycin turned yellow while those on 80 mg/L kanamycin turned brown with no regeneration observed at all the three concentrations (Figure 3).

**Table 1:** Effects of kanamycin on percentage of shoot regeneration from strawberry cv. Camarosa leaf explant after five weeks of culture. Means followed by the same letter (s) are not significantly different based on DMRT.

Kanamycin (mg/L)	0	10	20	30	40	50	60	70	80
Percentage of Regeneration (%)	100 ^a^	73 ^b^	33 ^c^	13 ^d^	6 ^de^	0 ^e^	0 ^e^	0 ^e^	0 ^e^

### 2.4. Optimization of Different Factors Affecting Transformation of Strawberry

The different factors tested in this study elicited significant differences on shoot regeneration of strawberry leaf explant after transformation. Shoot regeneration began by the end of week six and at the beginning of week seven after the inoculation of the leaf explant with *Agrobacterium* in the presence of timentin and cefotaxime, respectively. The highest percentage of putative transformants (83%) was obtained in the treatment with no preculture, bacterial OD_600_ of 0.6 and 150 mg/L cefotaxime (Table 2). The results showed that the leaf explant with no preculture and exposed to bacterial OD_600_ of 0.6 was more effective than OD_600_ of 1 in terms of mean number and percentage of shoot regeneration.

**Table 2:** Effects of preculture medium, bacterial OD_600_ and antibiotics on mean number and percentage of strawberry cv. Camarosa putative transformant production after eight weeks of culture. Means followed by the same letter(s) are not significantly different based on DMRT.

Parameters measured	Preculture		
	No Preculture	TDZ 16 µM	BAP 9 µM + TDZ 4 µM
**OD_600_ = 0.6 Cefotaxime 150 mg/L**			
Mean No.	6.7 ^b^	2.3 ^de^	6.3 ^b^
Percentage %	83 ^a^	23 ^ef^	67 ^cd^
**OD_600_ = 0.6 Timentin 300 mg/L**			
Mean No.	7.3 ^b^	3.7 ^cd^	1.7 ^e^
Percentage %	60 ^bc^	57 ^bcd^	17 ^fg^
**OD_600_ = 1 Cefotaxime 150 mg/L**			
Mean No.	4.7 ^c^	3.3 ^cd^	1 ^e^
Percentage %	43 ^d^	30 ^e^	7 ^g^
**OD_600_ = 1 Timentin 300 mg/L**			
Mean No.	4.3 ^c^	9.7 ^a^	7.3 ^b^
Percentage %	50 ^cd^	67 ^b^	50 ^b^

The highest mean number of shoots produced per leaf explant (9.7) was in the treatment with preculture on 16 µM TDZ, bacterial OD_600_ of 1 and 300 mg/L timentin. The percentage of shoot regeneration for this treatment was 67%. Meanwhile in the treatment with preculture on medium with 9 µM BAP plus 4 µM TDZ, bacterial OD_600_ of 0.6 and cefotaxime at 150 mg/L the highest percentage of shoot regeneration was also 67% (Table 2).

No shoot regeneration occurred in the negative control whereby all leaves became necrotic after two weeks of culture. The results of this study showed that high percentage and mean number of shoots per leaf explant of strawberry could be produced in treatments with no preculture. It is interesting to note that the mean number of shoots produced per explant was slightly higher in the presence of timentin while, the percentage of putative transformants was higher in the presence of cefotaxime. The lowest mean number of shoot regenerated per leaf explant (1) was in the presence of cefotaxime, bacterial OD_600_ of 1 and with preculture on medium with 9 µM BAP plus 4 µM TDZ.

The two tested antibiotics, timentin at 300 mg/L and cefotaxime at 150 mg/L, could completely remove the growth of bacteria surrounding the strawberry leaf explant. In response to cefotaxime the leaves turned brown more quickly than in response to timentin. Initially some area of the leaf explants turned brown on the second week of culture and subsequently shoots started to form on the adaxial side of the leaves at week 7.

### 2.5. Luciferase Reporter Gene Assay

The insertion of T-DNA into the strawberry plant genome was investigated using the luciferase reporter gene assay. The RLU per milligram fresh weight of putative kanamycin resistant strawberry plantlets in each treatment (Table 3) was different from the non-transformed control plant. Some plants did not show luciferase activity and their RLU values were 0 similar to non-transformed plants as a control.

**Table 3:** RLU of putative kanamycin resistant strawberry cv. Camarosa (mean of 10 plantlets).

Preculture	OD_600_ = 0.6		OD_600_ = 1	
	Cefotaxime 150 mg/L	Timentin 300 mg/L	Cefotaxime 150 mg/L	Timentin 300 mg/L
No preculture	14	24	18	23
TDZ 16 µM	15	18	23	12
BAP 9 µM + TDZ 4 µM	17	19	12	25

### 2.6. Nested PCR

**Figure 4:**
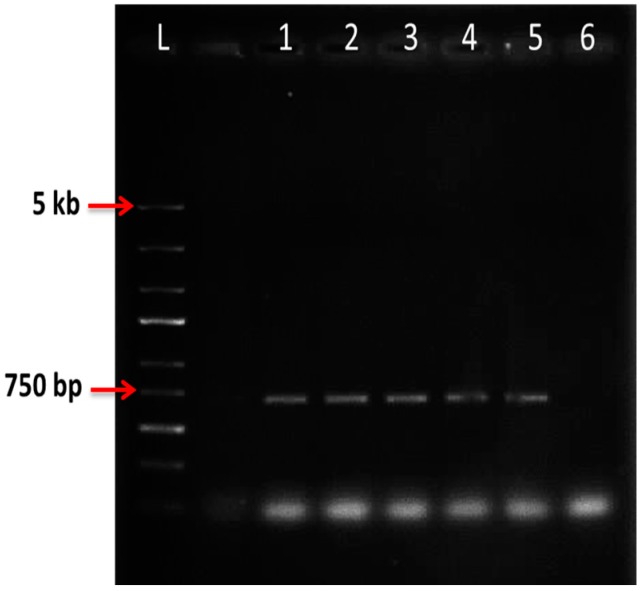
Amplification of PCR product in independent lines of putatively transformed strawberry cv. Camarosa harbouring luciferase gene; L: DNA Ladder; 1–5: Independent lines of putative transformed strawberry; 6: Non-transformed control plant.

Screening for the transgene insertion into the strawberry genome was performed by nested PCR. Nested PCR amplification yielded the expected 700 bp band corresponding to the designed primer for luciferase reporter gene (Figure 4). The amplified band was not observed in non-transformed control plants. In addition, PCR was performed for the luciferase negative plants and the result of the PCR confirmed the absence of the transgene in the strawberry genome.

The efficiency of transformation was determined based on the positive PCR result for each treatment. The highest transformation frequency was 100% in treatment with no preculture, bacterial OD_600_ = 0.6 and medium contained 150 mg/L cefotaxime. Slightly lower percentage (93%) was obtained in treatment with 300 mg/L timentin, preculturing on medium with BAP 9 µM + TDZ 4 µM and OD_600_ = 1. The lowest percentage of transformation efficiency (13%) was in the medium with 150 mg/L cefotaxime, pre-culturing explants in medium containing BAP 9 µM + TDZ 4 µM and OD_600_ = 1 (Table 4).

**Table 4:** Percentage of transformation efficiency of strawberry cv. Camarosa obtained based on PCR positive results.

Preculture	OD_600_ = 0.6		OD_600_ = 1	
	Cefotaxime 150 mg/L	Timentin 300 mg/L	Cefotaxime 150 mg/L	Timentin 300 mg/L
No preculture	100	60	40	53
TDZ 16 µM	33	53	20	67
BAP 9 µM + TDZ 4 µM	87	20	13	93

### 2.7. DNA Dot Blot Analysis

**Figure 5:**
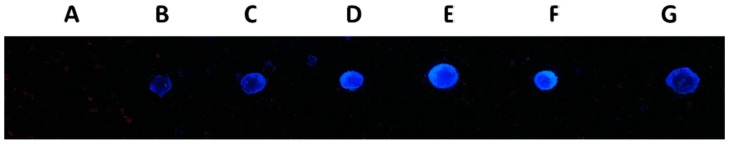
DNA dot blot analysis captured by camera; (**A**) Non-transformed control; (**B**–**G)** Transgenic lines of strawberry cv. Camarosa harbouring luciferase reporter gene.

DNA dot blot analysis of the transgenic strawberry plants showed hybridization signal with the probe against the luciferase gene (Figure 5). It confirmed the integration of LUC transgene into the genome of the transformed strawberry. However no signal was observed for any of the control plants.

### 2.8. Discussion

The efficiency of shoot regeneration after transformation was shown to be affected by various factors such as the binary vector used, application of potent plant growth regulators and antibiotics to the culture medium at different stages of the procedure, preculture, *Agrobacterium* strain and bacterial optical density (OD). Considerable numbers of studies have demonstrated *Agrobacterium*-mediated transformation of strawberry using callus and leaf explants [2] and that the callus culture system may increase the risk of somaclonal variation occurring.

In this study, high frequency shoot regeneration from leaf explant of strawberry cv. Camarosa was attained in the presence of TDZ. TDZ, a thidiazole-substituted phenyl urea growth regulator, is one of the potent cytokinins with the ability to induce cytokinin and auxin-like effects in plant tissues [5] which has been used for shoot regeneration of diploid [21] and octoploid [21,22,23] strawberries. The impressive effects of TDZ on shoot regeneration had been reported from various explants such as sepals of strawberry cv. Bounty [23], expanded leaves of strawberry cv. Toyonaka [24] or leaf lamina [25]. The results obtained in this study confirmed the potential of TDZ at 16 µM to promote high efficiency of shoot regeneration up to 100% and mean number of 24 shoots per leaf explant after 5 weeks of culture. In contrast, a lower regeneration percentage of 85% at 18.16 µM TDZ after eight weeks of culture was reported by Hussaini and Abdin [26].

Sensitivity of plants to antibiotics depends on the plant species, growth conditions and type of explant [8,27,28,29,30,31]. The incorporation of antibiotics for elimination of bacteria and selection of putative transformants usually results in the production of weak and albino shoots and likewise lower efficiency of transformation. Therefore, studies on the effects of different antibiotics on shoot regeneration from various explants are important in order to determine an antibiotic that can effectively control *Agrobacterium* with the least phytotoxic effects on growth at the post-infection stage.

The results from this study showed the superior effect of timentin on shoot regeneration of strawberry cv. Camarosa leaf explant among the antibiotics tested. Increasing the concentration of timentin from 150 to 300 mg/L showed significant increases in the mean number and percentage of shoot regeneration. It confirmed the potential of timentin to confer shoot regeneration ability with its hormone like effects. Timentin is a mixture of ticarcillin (96%) and clavulanic acid (4%). Ticarcillin is a penicillin-type antibiotic that works by stopping the growth of bacteria and clavulanate potassium is an enzyme inhibitor (beta-lactamase inhibitor) that helps the ticarcillin to work better. Finstad and Martin [32] demonstrated that shoots regenerated in the presence of timentin were healthier and vigorous compared to shoots regenerated in medium with carbenicillin. Increase in transformation frequency from leaf explant of strawberry cultured in medium with timentin was reported for strawberry cv. Totem [32] and cv. Chandler [33].

Minimal toxicity and plant hormone like activity of antibiotics were reported in the presence of carbenicillin and cefotaxime on most plant tissues. Nevertheless in this study cefotaxime exhibited lower phytotoxicity on shoot regeneration in comparison to carbenicillin. Cefotaxime a semi-synthetic, broad spectrum and cephalosporin antibiotic, is a β-lactam antibiotic that inhibits cell wall synthesis in dividing bacterial cells and results in cell lysis. Enhancement of shoot regeneration frequency by cefotaxime has been reported in other plants such as finger millet [34], wheat [35], pearl millet [36], sorghum [37], chilli [38], rice [28] and sugarcane [39].

Kaur *et al.* [39] reported that cefotaxime can improve frequency of transformation by killing the *Agrobacterium* after co-cultivation and improving the growth of regenerated shoots. However, Qin *et al*. [8] found that the application of carbenicillin either individually or in combination with cefotaxime was more effective than cefotaxime alone on shoot regeneration from leaf explant of strawberry cv. Toyonoka. The adverse effects of carbenicillin observed in this study for strawberry cv. Camarosa were the formation of callus and low efficiency of shoot regeneration per leaf explant. Carbenicillin is a semi-synthetic analogue of the naturally occurring benzyl-penicillin with auxin-like structural features [40] which breaks down to physiologically active levels of the auxin phenylacetic acid in culture medium [41]. This auxin-like effect of carbenicillin could be the cause of callus formation.

Ampicillin is also a beta-lactam antibiotic, belonging to the penicillin group, which differs from penicillin only by the presence of an amino group. Obembe *et al.* [42] demonstrated that ampicillin may be an effective, cheaper alternative compared to cefotaxime and timentin, in suppressing *Agrobacterium*. In spite of these good features of ampicillin the results from this study did not show its effectiveness in comparison to timentin and cefotaxime.

Overall, all tested antibiotics in this study were able to promote root formation from the regenerated shoots. This may be related to the plant hormone like activity of these antibiotics on plant tissues. Alternatively, since the antibiotics were assessed in combination with TDZ which has the ability to cause cytokinin and auxin-like effects on plant tissues arising from the induction of endogenous auxin, ethylene and cytokinin [43], the root formation may be related to the interaction of these antibiotics and this characteristic of TDZ.

Following antibiotic suppression of *Agrobacterium*, the clean explants subsequently need to be transferred to culture medium containing selectable marker antibiotics. Effective selection, using suitable selectable marker genes, can lead to a substantial reduction in the number of untransformed regenerants [42]. Most binary vectors used for transformation of strawberry carry the *nptII* gene conferring kanamycin resistance [10,33,44,45,46]. Based on the literature the possible doses of kanamycin that have been applied for selection of putative transformants from leaf explant of strawberry ranged from 25 mg/L [47,48] to 50 mg/L [33,49]. However, the lethal dosage of kanamycin is very genotype and explant-type dependent. Kanamycin at 150 mg/L needed to be applied for the selection of putative transformants in octoploid *Fragaria* cultivar Calypso [50] indicating the high resistance of the octoploid to kanamycin [49].

Reports have shown that improvement of transformation efficiency can be achieved by culturing tissues on preculture medium prior to transformation which makes the cells competent for regeneration and transgene integration. However, the results of this study showed that the highest percentage of putative transformants was achieved in treatment with no preculture. On the contrary, preculture in medium with TDZ showed slightly higher transformation efficiency compared to medium containing a combination of TDZ and BAP. In culture medium TDZ is more stable and active than BAP [51] which perhaps was the reason for the higher regeneration of shoot and transformation efficiency. Landi *et al.* [25] reported that TDZ was able to increase the possibility of shoot regeneration following inoculation with *Agrobacterium*. Despite the notable effects of TDZ to promote high number and percentage of shoot regeneration it however inhibited shoot elongation. In this study in order to overcome the problem of stunted shoots GA_3_ was added to the medium. Inhibition of shoot elongation by TDZ was also reported by Marcotrigiano *et al.* [52] in cranberry and Huetteman and Preece [53] in woody species. The negative effect of TDZ on shoot elongation in strawberry was removed by transferring the shoots to a medium with 4 mM zeatin [23].

The *Agrobacterium* strain is another factor potentially influencing the efficiency of genetic transformation. LBA4404 is the most common strain of *A. tumefaciens* that has been used so far for transformation of strawberry. The growth of *Agrobacterium* was strongly inhibited in treatments with cefotaxime. Channappagoudar [38] reported that cefotaxime was the most effective antibiotics against LBA4404. Nonetheless, the results of this study revealed that timentin effectively removed the bacteria from cultured leaf explant of strawberry and at the same time induced more number of shoots per explant. This improved regeneration could be due to timentin possessing less phytotoxicity effect than cefotaxime. However, Folta *et al*. [22] reported that timentin slightly reduced the growth and differentiation of strawberry shoot, but it still remained effective in curbing bacterial growth.

Based on the results of this study at lower bacterial OD_600_ of 0.6 and without preculture, high percentage of putative transformants (83%) and mean number of shoots (6.7) were obtained compared to the rest of the treatments. At the same optical density, the transformation efficiency was 67% after preculturing of explant on medium with 9 µM BAP and 4 µM TDZ. On the contrary at optical density of 1 the highest transformation efficiency was 67% which was achieved from explants precultured on medium with 16 µM TDZ. The utmost optical density applied in strawberry transformation studies was OD_600_ 1 [33,45]. Vellicce *et al.* [54] applied an OD_600_ of 0.2 for inoculation of strawberry leaf explant in their study. Several studies demonstrated the co-cultivation time from two days to two weeks on selective medium. However co-cultivation for three days was mostly applied in several studies on strawberry [33,45,47,49].

The highest efficiency of putative transformants obtained in this study for strawberry cv. Camarosa was 83%. Recently, Nasri and Bahramnejad [55] observed 2, 1 and 3% transformation efficiency for strawberry cvs. Camarosa, Paros and Kurdish, respectively. Compared to the result obtained in this study (83%), transformation percentage of 2% is very low. Husaini [33] achieved 41.25% transformation efficiency in strawberry cv. Chandler. Depending on the genotype and explant type, Alsheikh *et al.* [10] attained transformation efficiency ranging from 7.4% to 76.3% by calculating the percentage of explants that regenerated shoots on medium containing kanamycin.

In previous studies, the insertion and expression of transgenes into strawberry genome have been verified using reporter genes [10,48], PCR [4,32], Southern blot [4,32,56], northern blot [56,57,58] and ELISA [32]. The reporter genes that have been applied include β-glucuronidase (GUS or *uid*A), neomycin phosphotransferase (*nptII*), hygromycin phosphotransferase (*hptII*) and green fluorescence protein (GFP). Luciferase gene is another reporter gene with lack of endogenous enzyme activity in eukaryotic cells and it could even be detected in single transiently expressing cells [59]. It is a sensitive and accurate analysis where the result is proportional to the amount of functional luciferase enzyme.

A quick and reliable technique to confirm transformation is by PCR [60,61]. Nested PCR which is a very specific PCR amplification uses two pairs of primers in which the second primers are nested within the first pair of primers, hence giving the advantage of little or no contamination from non-specifically amplified PCR products from alternative primer target sequences. In this study nested PCR amplification of the luciferase gene amplicon in the strawberry plantlets confirmed the presence of the gene in the genome. Confirmation of transformation for strawberry cvs. Camarosa, Paros and Kurdish was performed by GUS assay and PCR of *uidA* reporter gene [55]. Wadl [49] applied multiplex PCR for amplification of the *nptII* and GFP genes in strawberry genome. The integration of AFP gene conferring freezing tolerance and *nptII* was confirmed by PCR analysis [62].

Different transformation efficiency have been reported in different strawberry cultivars. In this study the transformation efficiency of strawberry cv. Camarosa was attained based on PCR positive results. The highest percentage of transformation based on the PCR positive plantlets was 100% in treatment with no preculture and medium containing cefotaxime and OD_600_ = 0.6. The efficiency of transformation reported for petiole section of *Fragaria vesca* was 5% [63] while a 6.9% was achieved using leaf explant [48]. Various transformation efficiencies have been reported based on different factors affecting transformation. The selection of an appropriate antibiotic and *Agrobacterium* strain resulted in increased transformation efficiency of *F. vesca* L. to 15% as reported by Alsheikh *et al.* [10]. Further endeavors by Oosumi *et al.* [64] resulted in 100% transformation efficiency for *F. vesca* by applying 13.3 µM BA and 1.2 µM IBA in MS basal salt medium for shoot regeneration. In strawberry cv. Pajaro a transformation efficiency of 6.6% was recorded by Vellicce *et al.* [54]. DNA dot blot was performed and positive signals after hybridization of the genomic DNA with the probe were detected. The method provides advantages of rapid, easy and convenient analysis [65]. Overall application of different binary vector, no preculture, lower optical density and lower concentration of antibiotics might be some of the reasons to obtain high efficiency of transformation (83%), in comparison to the 2% recently achieved by Nasri and Bahramnejad [55].

## 3. Experimental Section

### 3.1. Binary Vector and Agrobacterium

*Agrobacterium tumefaciens* LBA4404 was transformed using the dual vector system: pGreen0049/pSoup by the freeze-thaw method [66]. The pGreen0049 binary vector and the pSoup vector from the pGreen vector series were obtained from the John Innes Centre, Norwich, UK. The pGreen0049 binary vector carries the plant selectable marker gene kanamycin phosphotransferase (*nptII*) controlled by the nopaline synthase (nos) promoter, bacterial selectable marker *nptII* gene, and luciferase (LUC) reporter gene driven by the CaMV 35S promoter on the T-DNA region. The pSoup vector carries the RepA function and tetracycline resistance gene as an antibiotic selectable marker.

#### 3.1.1. Effects of Different Concentrations of TDZ on Shoot Regeneration of Strawberry Leaves

Six-week-old trifoliate leaf explants of strawberry cv. Camarosa with the sides removed and incisions made on the leaf surface were cultured on MS [67] basal salts and vitamins supplemented with 0, 4, 8, 12, 16, 24 and 32 µM TDZ. The medium was poured into 250 mL flasks and each flask contained two explants. The leaf explants were placed with the abaxial side downwards on the medium. All treatments were repeated three times with 10 explants per treatment per replication. The percentage of explants producing shoots and the mean number of shoots produced per explant were recorded after five weeks of culture.

#### 3.1.2. Effects of Different Antibiotics on Shoot Regeneration from Strawberry Leaves

The effects of four different antibiotics, timentin, cefotaxime, carbenicillin and ampicillin were investigated. Leaf explants of strawberry were cut and cultured on MS basal salts and vitamins containing 16 µM TDZ and 150, 300 and 400 mg/L of each antibiotic individually. MS medium containing 16 µM TDZ was used as a control. The medium was dispensed into 250 mL flasks and each flask contained two explants. All treatments were repeated three times with 10 explants per treatment per replication. The percentage of shoot regeneration and the mean number of shoots produced per explant were recorded after five weeks of culture.

#### 3.1.3. Minimum Inhibitory Concentration of Kanamycin on Leaf Explants of Strawberry

The minimum inhibitory concentration (MIC) test was carried out by culturing leaf explants of strawberry with the sides removed and incisions made on the leaf surface on MS medium containing 16 µM TDZ and kanamycin at 0, 10, 20, 30, 40, 50, 60, 70 and 80 mg/L. The medium was dispensed into 250 mL flasks and each flask contained two explants. All treatments were repeated three times with 10 explants per treatment per replication. Subcultures were carried out at two-week intervals. The percentage of shoot regeneration was recorded after five weeks of culture.

#### 3.1.4. Optimization of Factors Affecting Transformation of Strawberry

In this study, 14 treatment combinations including two control treatments were performed to investigate the effects of preculture medium, bacterial optical density (OD) and antibiotics on bacterial elimination and shoot regeneration of putative transformants. Leaf explants of strawberry were cut and cultured on preculture media for four days prior to inoculation. The preculture media applied in this study were MS basal salt and vitamins supplemented with either 9 µM 6-benzylaminopurine (BAP) + 4 µM TDZ or 16 µM TDZ. In addition, leaf explant directly subjected to *Agrobacterium* inoculation without preculture was considered as one of the treatments. The preculture medium was dispensed into 100 × 25 mm Petri plates each containing 30 mL of medium and two leaf explants were cultured in each Petri plate. The leaf explants were inoculated with *Agrobacterium* at two optical densities (OD_600_) of 0.6 with colony forming units (CFU) equalling to 5 × 10^5^ cell/mL and OD_600_ of 1 with CFU equalling to 5 × 10^8^ cell/mL. The bacterial culture was diluted with liquid MSO medium (MS basal salts without sugar) at the ratio of 1:5 LB broth:liquid MSO.

Acetosyringone at a concentration of 100 µM was added to the bacterial culture at the time of infection. Leaf explants of strawberry were inoculated with *Agrobacterium* in 50 mL Falcon tube for 20 min and the leaves were blotted dry on autoclaved Whatman filter paper No. 1. After Inoculation the explants were returned to their preculture medium and kept in the dark for three days until the bacteria grew well and formed the holozoic surrounding the explant. For those treatments with no preculture the leaf explants were transferred to MS medium containing 16 µM TDZ immediately after inoculating with *Agrobacterium*. After three days, the explants were transferred to preselection medium which was MS basal salts and vitamins containing 16 µM TDZ and antibiotic (either 150 mg/L cefotaxime or 300 mg/L timentin) and kept under 16 h photoperiod condition for six days. The preselection medium was prepared in 100 × 25 mm Petri plates each containing 30 mL of medium and two leaf explants were placed per Petri plate. In the next step, the explants were transferred to selection medium consisting of MS basal salts and vitamins with 16 µM TDZ, 50 mg/L kanamycin and either 150 mg/L cefotaxime or 300 mg/L timentin to kill the bacteria. The medium was dispensed into 250 mL flasks and two leaf explants were cultured per flask. The explants were subcultured to fresh medium of the same constituents at two-week intervals for eight weeks. However, after the first subculture, kanamycin was omitted from the medium since strawberry leaves are sensitive to the antibiotic. In addition, leaf explants of strawberry were cultured on MS basal salts and vitamins with either 150 mg/L cefotaxime or 300 mg/L timentin and 50 mg/L kanamycin without *Agrobacterium* inoculation as a negative control. All treatments were repeated three times with 10 explants per treatment per replication. Finally the percentage and the mean number of putatively transformed explants were recorded for all the treatments. The percentage of putatively transformed explants for each treatment was calculated as bellow:

Percentage of putatively transformed explants (%) = Number of explants producing shoot × 100%/Total number of explants infected with *Agrobacterium*

### 3.2. Culture Condition

All cultures were maintained at 25 ± 1 °C under 16 h photoperiod with light intensity of 40 µmol m^−2^ s^−1^ provided by cool white fluorescent tubes.

#### 3.2.1. Statistical Analysis

Statistical analysis were performed using SAS (version 9.0; SAS Institute, Cary, NC, USA) and SPSS (version 16.1; Inc., Chicago, IL, USA) softwares. Data were subjected to one way analysis of variance (ANOVA) for mean comparison and significant differences were calculated according to Duncan multiple range test. Differences at *p* ≤ 0.05 were considered statistically significant.

#### 3.2.2. Luciferase Reporter Gene Assay

In 10 plantlets of every individual transformant and *in vitro* derived non-transformed plantlets as control, the fourth leaf of were collected and ground with liquid nitrogen in 2 mL Eppendorf tubes using a micro mortar. The ground tissue was mixed thoroughly with 200 µL of extraction buffer containing 100 mM potassium phosphate pH 7.0, 1 mM dithiothreitol and 1 mg/mL bovine albumin (Sigma-Aldrich Co. St, Louis, MO, USA). The mixture was kept at room temperature for 20 min and then centrifuged at 12,000 rpm for 5 min at 4 °C. The supernatant was transferred to new tubes. In the next step 120 µL of extract mixture was mixed with 120 µL luciferase substrate (One-glo Luciferase Assay System, Promega Corporation, Madison, WI, USA). Relative Luminescence Unit (RLU) values for luciferase activity in the leaf tissue were measured after 30 min using a TECAN microplate luminometer.

#### 3.2.3. Nested PCR Analysis of Putatively Transformed Strawberry Plantlets

Genomic DNA was isolated from each independent line of putative transformant and *in vitro* derived non-transformed plantlet as control using DNeasy Plant Mini Kit (QIAGEN, QIAGEN Inc., Hilden, Germany). PCR amplification was performed using a specific pair of forward and reverse primers designed based on the sequence of the luciferase gene to amplify the expected product of 700 bp (Table 5). The first PCR reaction was performed in 25 µL reaction volume containing 500 ng genomic DNA and Green Dream Taq Master Mix (Fermentas Inc., Hanover, NH, USA). The nested PCR was carried out by replacing the genomic DNA with 1 µL of the first PCR product. Similar PCR program was performed for the first and nested PCR following 5 min pre-denaturation at 95 °C and subsequent 35 cycles of 95 °C for 60 s, appropriate temperature for each primer for 60 s, 72 °C for 1 min/1 kb and a final extension step at 72 °C for 7 min. The PCR products were separated by electrophoresis on 1% agarose gel and after staining with ethidium bromide visualized with UV transilluminator. The percentage of transformation efficiency was calculated as below:

Percentage of transformation efficiency (%) = Number of plants producing the PCR fragments × 100%/Total number of plants tested with PCR

**Table 5:** Primers designed for nested PCR analysis of genomic DNA of putative transformants.

Primer	Forward Primer Sequence	Reverse Primer Sequence	Temperature (°C)	Size of Amplified Fragment (bp)
First primer pairs	5' TCTTCGCCAAAAGCACTCTGA 3'	5' TGGTGATTTCAGCGTACCGAATTA 3'	60	858
Nested primer pairs	5' CCTCTTTCGAAAGAAGTCGGGG 3'	5' ACAATTTGGACTTTCCGCCCT 3'	57.4	700

#### 3.2.4. DNA Dot Blot Analysis of the Transgenic Strawberry Lines

Dot blot analysis of the transgenic strawberry lines and non-transformed control plant were performed using DIG High Prime DNA Labeling and Detection Starter Kit II (Roche, Berlin, Germany). Five microgram of the extracted genomic DNA was applied as a spot on a nylon membrane. DNA fixation was achieved by washing the nylon membrane in 2× SSC solutions and baking the membrane at 120 °C for 30 min. The blot was hybridized with digoxigenin labeled luciferase probe at 42 °C followed by immunological detection. The blue dots (chemiluminescence) were captured after exposure to a Canon camera for 2–4 min at 25 °C in a dark room.

## 4. Conclusions

Strawberry, a member of the Rosaceae family, is considered a valuable horticultural crop for basic genomics and recombinant DNA studies because of its rapid growth and small genome compared to other woody species of the family. In addition, manipulation of octoploid strawberry plants with genes related to environmental stresses, diseases and phytopathogenic organisms such as pests, insects, fungi, bacteria, viruses and nematodes is of interest to produce profitable products. Hence the efficient protocol established in this study could be applicable for further genetic engineering of the Rosaceae family, specifically of strawberry plants.

## References

[1] Biswas M., Dutt M., Roy U., Islam R., Hossain M. (2009). Development and evaluation of *in vitro* somaclonal variation in strawberry for improved horticultural traits. Sci. Hortic..

[2] Mezzetti B. (2009). GMO strawberry: methods, risk and benefits. Genet Genom Rosaceae.

[3] Qin Y., Teixeira da Silva J.A., Zhang L., Zhang S. (2008). Transgenic strawberry: State of the art for improved traits. Biotechnol. Adv..

[4] Qin Y., Zhang S. (2007). Factors influencing the efficiency of *Agrobacterium*-mediated transformation in strawberry cultivar *Toyonaka*. J. Nucl. Agric. Sci..

[5] Folta K.M., Dhingra A. (2006). Invited review: Transformation of strawberry: The basis for translational genomics in Rosaceae. In Vitro Cell. Dev. Biol.-Plant.

[6] Barcelo M., El-Mansouri I., Mercado J.A., Quesada M.A., Pliego Alfaro F. (1998). Regeneration and transformation via *Agrobacterium tumefaciens* of the strawberry cultivar Chandler. Plant Cell Tiss Org. Cul..

[7] Gruchała A., Korbin M., Żurawicz E. (2004). Conditions of transformation and regeneration of Induka’ and Elista’ strawberry plants. Plant Cell Tiss Org. Cul..

[8] Qin Y., Teixeira da Silva J.A., Bi J., Zhang S., Hu G. (2011). Response of *in vitro* strawberry to antibiotics. Plant Growth Regul..

[9] Tang J., Liang S., Zhang J., Gao Z., Zhang S. (2009). pGreen-S: A clone vector bearing absence of enhanced green fluorescent protein for screening recombinants. Anal. Biochem..

[10] Alsheikh M., Suso H.P., Robson M., Battey N., Wetten A. (2002). Appropriate choice of antibiotic and *Agrobacterium* strain improves transformation of antibiotic-sensitive *Fragaria vesca* and *F. v. semperflorens*. Plant Cell Rep..

[11] Ieamkhang S., Chatchawankanphanich O. (2005). Augmentin as an alternative antibiotic for growth suppression of *Agrobacterium* for tomato (*Lycopersicon esculentum*) transformation. Plant Cell Tiss Org. Cul..

[12] Karami O., Esna-Ashari M., Karimi Kurdistani G., Aghavaisi B. (2009). *Agrobacterium*-mediated genetic transformation of plants: The role of host. Biol. Plantarum..

[13] Břiza J., Pavingerová D., Přikrylová P., Gazdova J., Vlasák J., Niedermeierová H. (2008). Use of phosphomannose isomerase-based selection system for *Agrobacterium*-mediated transformation of tomato and potato. Biol. Plantarum..

[14] Cho M.A., Moon C.Y., Liu J.R., Choi P.S. (2008). *Agrobacterium*-mediated transformation in *Citrullus lanatus*. Biol. Plantarum..

[15] Cheng M., Lowe B.A., Spencer T.M., Ye X., Armstrong C.L. (2004). Factors influencing *Agrobacterium*-mediated transformation of monocotyledonous species. In Vitro Cell. Dev. Biol.-Plant.

[16] Olhoft P.M., Flagel L.E., Donovan C.M., Somers D.A. (2003). Efficient soybean transformation using hygromycin B selection in the cotyledonary-node method. Planta.

[17] Wu H., Sparks C., Amoah B., Jones H. (2003). Factors influencing successful *Agrobacterium*-mediated genetic transformation of wheat. Plant Cell Rep..

[18] Zambre M., Terryn N., de Clercq J., de Buck S., Dillen W., van Montagu M., van der Straeten D., Angenon G. (2003). Light strongly promotes gene transfer from *Agrobacterium tumefaciens* to plant cells. Planta.

[19] Salas M., Park S., Srivatanakul M., Smith R. (2001). Temperature influence on stable T-DNA integration in plant cells. Plant Cell Rep..

[20] Hellens R.P., Edwards E.A., Leyland N.R., Bean S., Mullineaux P.M. (2000). pGreen: A versatile and flexible binary Ti vector for *Agrobacterium*-mediated plant transformation. Plant Mol. Biol..

[21] Wang W., Vinocur B., Shoseyov O., Altman A. (2004). Role of plant heat-shock proteins and molecular chaperones in the abiotic stress response. Trends Plant Sci..

[22] Folta K.M., Dhingra A., Howard L., Stewart P.J., Chandler C.K. (2006). Characterization of LF9, an octoploid strawberry genotype selected for rapid regeneration and transformation. Planta.

[23] Debnath S.C. (2005). Strawberry sepal: Another explant for thidiazuron-induced adventitious shoot regeneration. In Vitro Cell. Dev. Biol.-Plant.

[24] Qin Y., Shanglong Z., Asghar S., Lingxiao Z., Qiaoping Q., Kunsong C., Changjie X. (2005). Regeneration mechanism of Toyonoka strawberry under different color plastic films. Plant Sci..

[25] Landi L., Mezzetti B. (2006). TDZ, auxin and genotype effects on leaf organogenesis in Fragaria. Plant Cell Rep..

[26] Husaini A.M., Abdin M.Z. (2008). Development of transgenic strawberry (*Fragaria x ananassa* Duch.) plants tolerant to salt stress. Plant Sci..

[27] Petri C., Lopez-Noguera S., Alburquerque N., Egea J., Burgos L. (2008). An antibiotic-based selection strategy to regenerate transformed plants from apricot leaves with high efficiency. Plant Sci..

[28] Grewal D., Gill R., Gosal S.S. (2006). Influence of antibiotic cefotaxime on somatic embryogenesis and plant regeneration in indica rice. Biotechnol. J..

[29] Aoshima Y. (2005). Efficient embryogenesis in the callus of tea (*Camellia sinensis*) enhanced by osmotic stress or antibiotic treatment. Plant Biotechnol..

[30] Teixeira da Silva J.A.T. (2003). Filter paper type affects the morphogenic programs and buffers the phytotoxic effect of antibiotics in chrysanthemum and tobacco thin cell layers. HortScience.

[31] Teixeira da Silva J.A.T., Fukai S. (2001). The impact of carbenicillin, cefotaxime and vancomycin on chrysanthemum and tobacco TCL morphogenesis and *Agrobacterium* growth. J. Appl. Hortic..

[32] Finstad K., Martin R.R. (1995). Transformation of strawberry for virus resistance. Acta Hortic..

[33] Husaini A.M. (2010). Pre-and post-agroinfection strategies for efficient leaf disk transformation and regeneration of transgenic strawberry plants. Plant Cell Rep..

[34] Eapen S., George L. (1990). Influence of phytohormones, carbohydrates, amino acids, growth supplements and antibiotics on somatic embryogenesis and plant differentiation in finger millet. Plant Cell Tiss Org. Cul..

[35] Borrelli G., di Fonzo N., Lupotto E. (1992). Effect of cefotaxime on callus culture and plant regeneration in durum wheat. J. Plant Physiol..

[36] Plus J., George L., Eapen S., Rao P. (1993). Enhanced plant regeneration in pearl millet (*Pennisetum americanum*) by ethylene inhibitors and cefotaxime. Plant Cell Tiss Org. Cul..

[37] Rao A., Sree K.P., Kishor P.B.K. (1995). Enhanced plant regeneration in grain and sweet sorghum by asparagine, proline and cefotaxime. Plant Cell Rep..

[38] Channappagoudar S.B. (2007). Studies on *In Vitro* Regeneration and Genetic Transformation in Chilli (*Capsisum annuum* L.). Ph.D. Thesis.

[39] Kaur A., Gill M., Ruma D., Gosal S. (2008). Enhanced *in vitro* shoot multiplication and elongation in sugarcane using cefotaxime. Sugar Technol..

[40] Costa M., Nogueira F., Figueira M., Otoni W., Brommonschenkel S., Cecon P. (2000). Influence of the antibiotic timentin on plant regeneration of tomato (*Lycopersicon esculentum* Mill.) cultivars. Plant Cell Rep..

[41] Holford P., Newbury H. (1992). The effects of antibiotics and their breakdown products on the *in vitro* growth of *Antirrhinum majus*. Plant Cell Rep..

[42] Obembe O., Adesoye A., Machuka J. (2005). Evaluation of the effectiveness of selected antibiotics in the suppression of *Agrobacterium* from Cowpea (*Vigna unguiculata* L. Walp.) embryo explants and as potential selective agents in *Agrobacterium*-mediated transformation. J. Trop. Microbiol. Biotechnol..

[43] Murthy B., Singh R., Saxena P.K. (1996). Induction of high-frequency somatic embryogenesis in geranium (*Pelargonium x hortorum* Bailey cv. Ringo Rose) cotyledonary cultures. Plant Cell Rep..

[44] Jiménez-Bermúdez S., Redondo-Nevado J., Muñoz-Blanco J., Caballero J.L., López-Aranda J.M., Valpuesta V., Pliego-Alfaro F., Quesada M.A., Mercado J.A. (2002). Manipulation of strawberry fruit softening by antisense expression of a pectate lyase gene. Plant Physiol..

[45] Dhir S., Hinchee M.A.W., Layton J.G., Oakes J.V. (2001). Methods for Strawberry Transformation Using *Agrobacterium Tumefaciens*. U.S. Patent.

[46] Cordeno de Mesa C., Jiménez-Bermúdez S., Pliego-Alfaro F., Quesada M.A., Mercado J.A. (2000). *Agrobacterium* cells as microprojectile coating: a novel approach to enhance stable transformation rates in strawberry. Funct Plant Biol..

[47] Mathews H., Dewey V., Wagoner W., Bestwick R.K. (1998). Molecular and cellular evidence of chimaeric tissues in primary transgenics and elimination of chimaerism through improved selection protocols. Transgenic Res..

[48] El-Mansouri I., Mercado J.A., Valpuesta V., López-Aranda J.M., Pliego-Alfaro F., Quesada M.A. (1996). Shoot regeneration and *Agrobacterium*-mediated transformation of *Fragaria vesca* L.. Plant Cell Rep..

[49] Wadl P.A. (2006). Improved Regeneration and Agrobacterium-Mediated Transformation of Wild Strawberry (*Fragaria vesca* L.). Master’s Thesis.

[50] Schaart J.G., Krens F.A., Pelgrom K.T.B., Mendes O., Rouwendal G.J.A. (2004). Effective production of marker-free transgenic strawberry plants using inducible site-specific recombination and a bifunctional selectable marker gene. Plant Biotechnol. J..

[51] Fahmy A.H., El-Shafy Y.H., El-Shihy O.M., Madkour M. (2006). Highly efficient regeneration system via somatic embryogenesis from immature embryos of Egyptian wheat cultivars (*Triticum aestivum* L.) using different growth regulators. World J. Agr. Sci..

[52] Marcotrigiano M., McGlew S.P., Hackett G., Chawla B. (1996). Shoot regeneration from tissue-cultured leaves of the American cranberry (*Vaccinium macrocarpon*). Plant Cell Tiss Org. Cul..

[53] Huetteman C.A., Preece J.E. (1993). Thidiazuron: A potent cytokinin for woody plant tissue culture. Plant Cell Tiss. Org. Cul..

[54] Vellicce G.R., Ricci J.C.D., Hernández L., Castagnaro A.P. (2006). Enhanced resistance to *Botrytis cinerea* mediated by the transgenic expression of the chitinase gene ch5B in strawberry. Transgenic Res..

[55] Nasri S., Bahramnejad B. (2013). Regeneration and Agrobacterium-mediated transformation of three economically important strawberry cultivars Kurdistan, Camarosa and Paros. Prog. Biol. Sci..

[56] Morgan A., Baker C.M., Chu J.S.F., Lee K., Crandall B.A., Jose L. (2002). Production of herbicide tolerant strawberry through genetic engineering. Acta Hortic..

[57] Chalavi V., Tabaeizadeh Z., Thibodeau P. (2003). Enhanced resistance to *Verticillium dahliae* in transgenic strawberry plants expressing a *Lycopersicon chilense* chitinase gene. J. Am. Soc. Hortic. Sci..

[58] Liu F., Guo Y., Gu D., Xiao G., Chen Z., Chen S. (1997). Salt tolerance of transgenic plants with BADH cDNA. Acta Genet Sin..

[59] Kost B., Schnorf M., Potrykus I., Neuhaus G. (1995). Non‐destructive detection of firefly luciferase (LUC) activity in single plant cells using a cooled, slow‐scan CCD camera and an optimized assay. Plant J..

[60] Liu C.W., Chen J.J.W., Kang C.C., Wu C.H., Yiu J.C. (2012). Transgenic lettuce (*Lactuca sativa* L.) expressing H1N1 influenza surface antigen (neuraminidase). Sci. Hortic..

[61] Goto F., Yoshihara T., Saiki H. (2000). Iron accumulation and enhanced growth in transgenic lettuce plants expressing the iron-binding protein ferritin. Theor. Appl. Genet.

[62] Firsov A., Dolgov S. (1999). Agrobacterial transformation and transfer of the antifreeze protein gene of winter flounder to the strawberry. Acta Hortic..

[63] Haymes K., Davis T. (1998). *Agrobacterium*-mediated transformation of “Alpine” *Fragaria vesca*, and transmission of transgenes to R1 progeny. Plant Cell Rep..

[64] Oosumi T., Gruszewski H.A., Blischak L.A., Baxter A.J., Wadl P.A., Shuman J.L., Veilleux R.E., Shulaev V. (2006). High-efficiency transformation of the diploid strawberry (*Fragaria vesca*) for functional genomics. Planta.

[65] Brown T. (2001). Dot and slot blotting of DNA. Current Protocols in Molecular Biology.

[66] An G., Ebert P.R., Mitra A., Ha S.B., Gelvin S.B., Schilperoort R.A. (1988). Binary vectors. Plant Molecular Biology Manual.

[67] Murashige T., Skoog F. (1962). A revised medium for rapid growth and bio assays with tobacco tissue cultures. Physiol. Plantarum..

